# The Zoom Picture Book Game: A Creative Way to Promote Teamwork in Undergraduate Medical Education

**DOI:** 10.7759/cureus.6964

**Published:** 2020-02-12

**Authors:** Xiao Chi Zhang, Arjun Balakumar, Carlos Rodriguez, Anthony Sielicki, Dimitrios Papanagnou

**Affiliations:** 1 Emergency Medicine, Thomas Jefferson University, Philadelphia, USA

**Keywords:** team building, gamification, undergraduate medical education

## Abstract

Effective team building and leadership are crucial in running an effective and safe healthcare system with improved patient care and clinical outcomes. Currently, there is a great demand for formal leadership training throughout the extensive medical education curriculum. We constructed an interactive team-building activity utilizing gamification-theory with the Zoom game. The Zoom activity requires a team of learners to organize a set of sequential images, each of which contains a “zoomed out” section from the previous image, into the correct order within a set time frame. Given the unique and approachable nature of this team-based activity, we propose the following: 1) to introduce the Zoom game as a team-building and communication fostering exercise in undergraduate medical education and 2) to assess baseline teamwork skills of first-year medical students through an immersive gaming experience. With this in mind, 260 first-year medical students (class of 2020) at an urban-city medical school were enrolled in the Zoom Team Building Activity as part of their orientation. The students were randomly assigned to 11 teams, comprising 23-24 students and two faculty facilitators per team and completed the activity in the allotted time frame. The average time to complete the Zoom game was 24 minutes, and all the teams successfully placed the pictures in the correct order. Facilitators noted that the Zoom game strongly encouraged friendly interactions, intercollegiate high values, mutual respect, confidence, and trust among each other. Students observed take-home points such as selecting a leader, designating specific roles, and encouraging closed-loop communication. Overall, the Zoom activity game is an interactive, fun, and easily accessible team-building and communication fostering exercise in undergraduate medical education. Further studies on the Zoom game exercise would be essential to determine whether it has a continuous and enduring effect on developing team building among medical students.

## Introduction

Team building and leadership are two critical features in running an effective and safe healthcare system. Studies have indicated that effective leadership correlates with improved patient care and clinical outcomes [1-6]. As medical students graduate to become resident physicians, they are tasked with newfound responsibilities of simultaneously managing their patient care, communicating treatment plans, and coordinating care from the nurses, techs, consultants, and supervising physicians. Despite robust scientific literature supporting the importance of effective teamwork and leadership in promoting student learning, healthcare performances, and improved patient outcomes, there is still a great demand for formal leadership training throughout the extensive medical education curriculum [7-9]. In order to address the leadership gap between resident and attending physicians, we posit that leadership and team-building training should begin at the undergraduate medical education setting, rather than delaying it until residency training. While teaching and leadership skills are formally taught during residency, we believe that junior learners, such as first-year medical students may benefit from interactive, innovative, and low-stake teaching strategies. A review of team building and leadership training suggests that retreats and other forms of dedicated leadership exercises have been shown to engage the learners and simultaneously improve leadership and teamwork skills [10,11].

The Zoom Team Building Activity is a well-regarded game based on a picture book, “Zoom”, by Istvan Banyai and repurposed as a team-building and leadership exercise [12,13]. The activity requires a team of 8-30 players to organize a set of 30 sequential images, each of which contains a “zoomed out” section from the previous image, into the correct order within 30 minutes. For example, a person is seen on a pool deck, which zooms out to be on a cruise ship, which again zooms out to be an advertisement for a cruise ship on a bus in sequential pictures. The caveat is that players are not allowed to look at other players’ pictures, but must describe the pictures’ contents among the team to determine the correct order. Once the final order is decided by the team, all of the players will display their pictures and compare their order with the correct sequence. Given the unique and approachable nature of this team-based activity, we propose the following: 1) to introduce the Zoom game as a team-building and communication fostering exercise in undergraduate medical education and 2) to assess baseline teamwork skills of first-year medical students through an immersive gaming experience.

## Technical report

Study setting

The Zoom activity was conducted at a conference room at the Sidney Kimmel Medical School, associated with Thomas Jefferson University, Philadelphia, PA. The conference-room setting allowed all players to move around the room with ease to communicate the details of their pictures.

Study-participant selection

Study inclusion criteria were as follows: 1) participants should be 18 years and older; 2) they should be first-year medical students attending Thomas Jefferson University. Individuals who were unable to consent (e.g., due to altered mental status, dementia, or developmental delay) were excluded. We did not anticipate community attendants to be intoxicated, incarcerated, or in clinical distress during this study. All 260 incoming first-year medical students were required to attend this activity as part of their mandatory orientation training. This study was deemed exempt from institutional review board (IRB) approval by the Jefferson IRB.

Materials/personnel requirements

In order to play the game, each group, consisting of 8-30 students, is required to cut out and separate a set of 30 pictures from the Zoom game (with one picture on every individual sheet). Two facilitators are responsible for organizing the activity, handing out the pictures, and evaluating the team performance.

Detailed activity description

Preparation (Five Minutes)

The facilitators first select the number of sequential pictures in play for the activity. The number of active pictures must be equal to the number of total players (a maximum of 30 pictures). If there are fewer than 30 players, all pictures must be in the correct sequence to avoid lapsing photographs. The picture order is then shuffled before distribution.

Introduction (Five Minutes)

The facilitators explain the Zoom activity to the group and then hand out the sequential pictures facedown to each participant. Each participant is given a few moments to thoroughly examine his or her pictures.

Activity (20 Minutes)

The group communicates with each other about the order they believe the pictures to be in without showing each other the pictures. The group is then instructed to put the pictures in order as efficiently as possible (with no information given about what the “order” is). Once they believe they have put the images in order, the images are revealed sequentially to see if the order is correct. If one image is not correct, the game continues until everything is in the correct order. Participants are not allowed to show each other their images but can demonstrate/describe their image through actions and words. The entire game is expected to last 30 minutes in total.

Debriefing (Five Minutes)

After the completion of the Zoom game, all players participate in a brief facilitator-led debriefing to discuss the overall observed teamwork during the activity. Players are encouraged to disclose take-home points of the activity.

Activity Evaluation

Following the end of the activity, the facilitators are expected to complete two separate, blinded evaluations of the participants’ leadership, preparation, participation, communication, and listening skills through the institutional Jefferson Teamwork Observation Guide (JTOG) [14]. The JTOG is a validated, user-friendly, 14-item Likert-scaled (1 = strongly agree, 4 = strongly disagree) evaluation instrument based on learner and clinician feedback for interprofessional education, primarily designed to assess teamwork [14].

Results

In our activity, 260 first-year medical students in the graduating class of 2022 at Sidney Kimmel Medical School of Thomas Jefferson University, Philadelphia, PA participated as part of their orientation. The students were randomly assigned to 11 teams, comprising 23-24 students and two faculty facilitators per team. The average time to complete the Zoom game was 24 minutes and all the teams successfully placed the pictures in the correct order. The mean JTOG scores were calculated based on the blinded assessment of each team’s two facilitators. Facilitators noted that the Zoom game strongly encouraged friendly interactions (Likert score: 1.2), intercollegiate high values (Likert score: 1.4), and mutual respect, confidence, and trust (Likert score: 1.4) among each other (Table 1). In contrast, areas of improvement were noted in the identification of a team leader (Likert score: 2.1), understanding the roles and responsibilities of each member (Likert score: 1.9), and the overall activity preparation (Likert score: 1.9). Comments captured from the JTOG for first-year medical students were evaluated through open-axial qualitative analysis performed by the authors, who have extensive experience with qualitative research (Table 2). According to the students, the primary take-home points from the Zoom activity centered on the following: 1) selecting a leader 2) designating specific roles 3) eliciting specific directions; 4) recognizing the challenges of macro- and micro-managing siloed teams, and 5) encouraging closed-loop communication.

**Table 1:** JTOG assessment of the Zoom activity for first-year medical students JTOG: Jefferson Teamwork Observation Guide; SD: standard deviation *1 = strongly agree, 4 = strongly disagree

JTOG assessment	Mean Likert score*	SD
There appeared to be a team leader that coordinated the discussion	2.1	0.7
The team leader facilitated the discussion rather than dominating it	1.7	0.6
Members of the team who came prepared to discuss the case/situation contributed to the discussion	1.9	0.6
Members of the team who were involved in the case/situation contributed to the discussion	1.5	0.5
Discussion points were distributed among all team members	1.8	0.7
Member of the team appeared to understand the roles and responsibilities of other members of the team	1.9	0.8
Team members appeared to have respect, confidence, and trust in one another	1.4	0.5
Team members listened and paid attention to each other	1.6	0.6
Team members listened to and considered the input of others before pressing their own ideas	1.8	0.6
Team members added other supporting pieces of information from their profession-specific perspective regarding the case/situation	1.6	0.7
The opinions of team members were valued by other members	1.4	0.5
Team members appeared to feel free to disagree openly with each other's ideas	1.8	07
Team members sought out opportunities to work with others on specific tasks	1.4	0.5
Team members engaged in friendly interaction with one another	1.2	0.4

**Table 2:** Themes developed from responses to JTOG assessment by the participants JTOG: Jefferson Teamwork Observation Guide

JTOG feedback	Themes
Positive impacts	Leadership: naturally emerging leadership, proper instructive techniques; calm; leader-requested debriefing
	Communication: asking for help, suggestions, and counter-arguments; frequent timeouts; acknowledging others; hand-raising.
	Organization: preferred one person in charge; initial delegation, using dry-erase boards
	Knowledge: quick pattern recognition
Negative Impacts	Location: small rooms and cluttered desk
	Leadership: ranging from too many leaders to absent leadership
	Communication: disruptive side conversation; interruptions; siloed communication between small groups
	Organization: tribal mentality; lack of initial system; resistant to movement
	Knowledge: lack of thought flexibility
Take-home points	Listeners can be leaders; teamwork; specificity is important; clear role definition; importance of micro- and macro-management

## Discussion

The investigators were successful in utilizing the Zoom game as an effective team building and leadership training activity and assessing the baseline teamwork skills for first-year medical students. The Zoom activity allowed first-year medical students to engage in a low-stake, controlled, interactive, and immersive activity that transcended their training, expertise, and educational background. Facilitators noted that the first-year medical students exemplified friendly and respectful intercollegiate skills, but were less adept at selecting leaders, assigning roles, and preparing for the activity challenges. The open-axial qualitative assessment of the JTOG response also demonstrated that the participants recognized the important aspects and challenges of team building and leadership. Additional common themes included a preference for larger rooms and recognizing the detrimental effects of disruptive side conversions, interruption, and lack of closed-loop communication.

In order to prepare medical students for residency, the Association of American Medical Colleges (AAMC) has issued a set of 13 guidelines and expectations, known as Core Entrustable Professional Activities (EPAs), for graduating medical students to learn, develop, and ultimately be able to perform without supervision by graduation [15]. In accordance with the AAMC Core EPAs, the Zoom game also affords the learners to be exposed to specific components of EPA9: collaborate as a member of an interprofessional team - interpersonal and communication skills (ICS2: work effectively with healthcare team; ICS3: communicate effectively with colleagues within the same profession), professionalism (P1: demonstrate compassion, integrity, and respect), and interprofessional collaboration (IPC1: work with other health professional to maintain mutual respect, dignity, and trust).

We recognize that there were several limitations to our study. This was a feasibility study at a single medical school, assessing the baseline teamwork skills for the first-year medical students during orientation. While there are numerous published studies on teaching teamwork in undergraduate medical education, most involve case scenarios or role-playing within a non-acute healthcare setting or simulation centers with high-fidelity mannequins [16-18]. Our study focused primarily on assessing and reflecting on unique aspects of teamwork and leadership of first-year medical students before they began their medical training, making it unique compared to previous studies. While the JTOG was a useful assessment tool, an additional test is warranted to see how the early implementation of the Zoom activity can affect the realization of EPA9 guidelines during the students' training period. Furthermore, due to a large number of students, we required a large number of facilitators to evaluate and supervise the students. Additionally, more iterations and advanced statistical software will be required to calculate inter-rater reliability between the facilitators' scores in the future.

## Conclusions

The Zoom activity game is an interactive and easily accessible team-building exercise that can be used to assess baseline teamwork skills of first-year medical students through an immersive gaming experience. By engaging first-year medical students in the Zoom game, we were able to highlight the aspects of teamwork necessary among the students to succeed as collaborative and supportive future physicians. Further studies on the Zoom game exercise would be essential to determine whether it can have a continuous and lasting effect on instilling team-building qualities among medical students.
